# Nanoporous Membranes of Densely Packed Carbon Nanotubes Formed by Lipid-Mediated Self-Assembly

**DOI:** 10.1021/acsabm.2c00585

**Published:** 2022-09-07

**Authors:** Martin Vögele, Jürgen Köfinger, Gerhard Hummer

**Affiliations:** † Department of Theoretical Biophysics, Max Planck Institute of Biophysics, Max-von-Laue-Str. 3, 60438 Frankfurt am Main, Germany; ‡ Institute for Biophysics, Goethe University Frankfurt, Max-von-Laue-Str. 1, 60438 Frankfurt am Main, Germany

**Keywords:** carbon nanotubes, membranes, lipids, bioinspired self-assembly, nanopores, diffusion

## Abstract

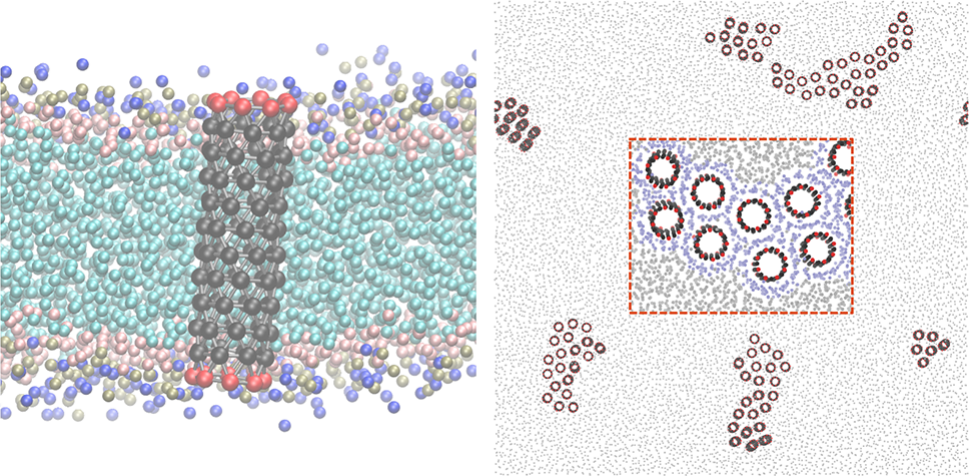

Nanofiltration technology faces the competing challenges of achieving high fluid flux through uniformly narrow pores of a mechanically and chemically stable filter. Supported dense-packed 2D-crystals of single-walled carbon nanotube (CNT) porins with ∼1 nm wide pores could, in principle, meet these challenges. However, such CNT membranes cannot currently be synthesized at high pore density. Here, we use computer simulations to explore lipid-mediated self-assembly as a route toward densely packed CNT membranes, motivated by the analogy to membrane-protein 2D crystallization. In large-scale coarse-grained molecular dynamics (MD) simulations, we find that CNTs in lipid membranes readily self-assemble into large clusters. Lipids trapped between the CNTs lubricate CNT repacking upon collisions of diffusing clusters, thereby facilitating the formation of large ordered structures. Cluster diffusion follows the Saffman-Delbrück law and its generalization by Hughes, Pailthorpe, and White. On longer time scales, we expect the formation of close-packed CNT structures by depletion of the intervening shared annular lipid shell, depending on the relative strength of CNT–CNT and CNT–lipid interactions. Our simulations identify CNT length, diameter, and end functionalization as major factors for the self-assembly of CNT membranes.

## Introduction

Carbon nanotubes (CNTs) are excellent water conductors down to subnanometer pore diameters.^1−5^ Aligned and hexagonally packed CNTs can form membrane-like structures.^6^ Such CNT membranes could be used for the design of dielectric materials^7^ and for filtration or desalination.^8−11^ The selectivity of the filter can be tuned by functional groups at the ends of the CNTs.^12,13^ CNT membranes have been built by growing forests of CNTs on a substrate and encapsulating them with silicon nitride,^3^ epoxy resins,^14^ or ceramics.^15^ However, the maximum possible pore density is not reached and the filled space between the CNTs does not contribute to solvent conduction. Here, we explore lipid-mediated self-assembly as an alternative route to densely packed CNT membranes.

As a key requirement, CNTs have been successfully introduced into lipid vesicles.^16−19^ CNTs can be pushed into or through lipid membranes,^20,21^ but they can also be internalized by passive diffusion^22−24^ or by growing bilayer structures around CNTs from dispersed solution.^25^ The amount of lipid coating plays a major role in the formation of transmembrane channels with nonfunctionalized CNTs.^26^ Coarse-grained as well as atomistic simulations^13,27^ have shown that the equilibrium orientation of open CNTs in a membrane strongly depends on their lengths and the chemical functionalization of their ends. Polar end-functionalizations keep the nanotube in an up-right orientation and prevent short pores from becoming blocked by lipid head groups.^13^

With CNT insertion being comparably well characterized, we concentrate here on the lipid-mediated CNT assembly into dense, two-dimensionally (2D) ordered structures as a route for the production of CNT membranes. CNT porins are open CNTs and share a cylindrical shape with barrel-shaped transmembrane proteins.^28^ We can therefore think of CNTs as biocompatible artificial pores in membranes. Depending on shape and hydrophobicity of their transmembrane domains, proteins can assemble into clusters and form densely packed two-dimensional crystals.^29^ CNT porins forming similarly ordered structures, with or without lipids retained on the outside, could be placed on top of a porous support, e.g., a polymeric mesh, to build a mechanically and chemically stable filter device with much higher pore density than CNT-containing membranes obtained by previous techniques.^3,14^ As an essential requirement for assembly, CNTs are mobile in lipid membranes, even for supported membranes as shown experimentally.^30,31^ Our focus is thus on the collective self-assembly process and the identification of the factors controlling spontaneous CNT membrane formation.

## Materials and Methods

In a series of molecular dynamics (MD) simulations, we studied the assembly of 100 initially dispersed CNTs differing in length, diameter, and functionalization state within membranes of different lipid compositions (see Tables S2–S7). Lipids were described using the coarse-grained Martini model,^32^ which has been extended to many other types of organic compounds.^27,33−35^ For the lipid types used in this work and their abbreviations, see Table S1. Martini simulations have already been used successfully to study the aggregation of proteins and nanoparticles in lipid membranes.^36−40^ We concentrated on membranes consisting of pure POPC lipids, which are common in biological membranes, biocompatible, and widely used in biophysical studies. However, below we study effects of different lipids. Additionally we used a Martini model for monoolein (MO) originally designed for simulations of in-meso protein crystallization.^41,42^ For the CNT porins, we used a model developed previously^13^ by adapting models for fullerenes^34^ and capped CNTs.^27^ Our model has already been used to study CNT-mediated fusion of lipid vesicles^19,25^ and CNTs in flat membranes.^13,43,44^ For more details on the simulations, see the Supporting Information. Parameter files and results are available on https://github.com/bio-phys/cnt-clusters.

## Results and Discussion

### Dynamics of Cluster Formation

**Figure 1:**
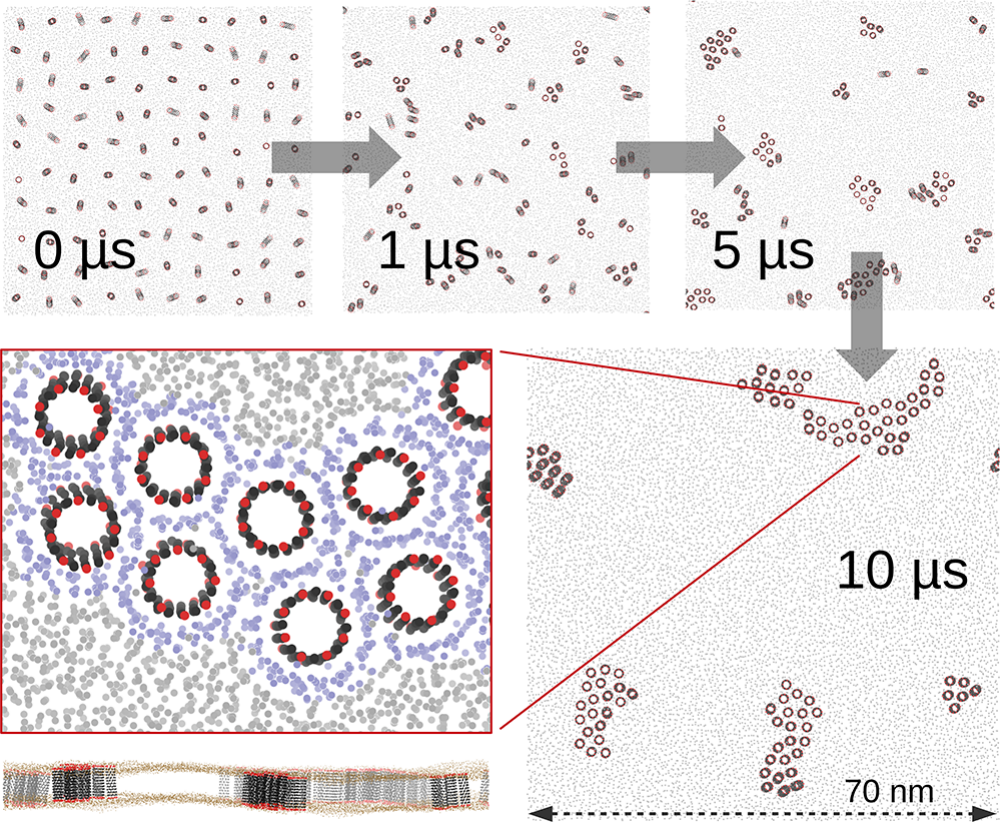
Cluster formation of CNT porins with polar functional end-groups in a POPC lipid bilayer. (Top) Time series of top views on the membrane containing 100 CNTs, with CNT carbon beads in black, polar functional groups in red, and lipid PO4 beads in gray. (Bottom) Zoom-in on CNT cluster with lipids in the first annular shell colored light purple. The lower left image shows a side view with lipid PO4 groups in brown.

**Figure 2:**
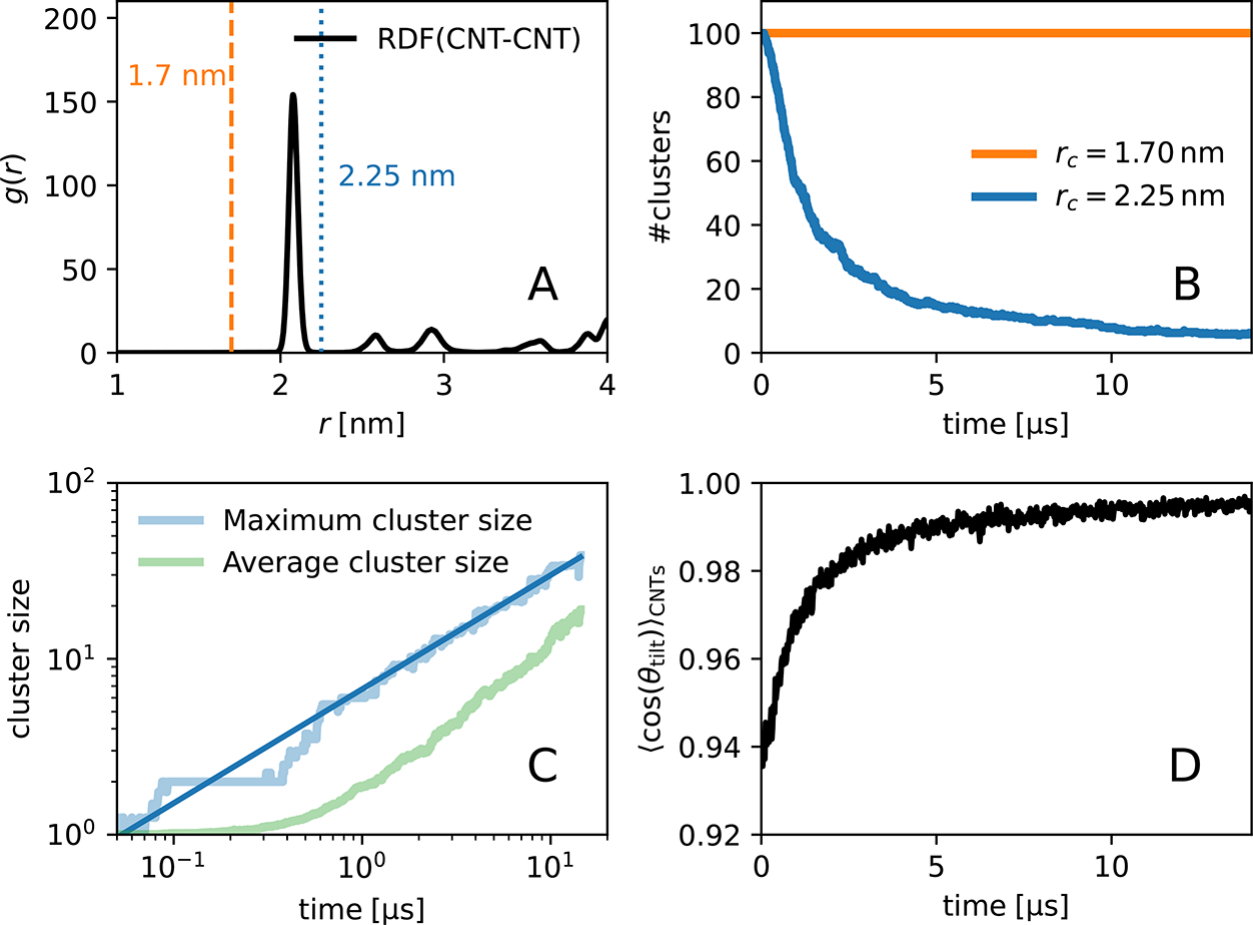
Clustering of CNT porins with polar functional end-groups from coarse-grained simulations containing 100 CNTs. (A) Radial distribution function of CNT–CNT center-of-mass distances. Vertical lines indicate the cutoff radii used to define CNT clusters with full contact (1.7 nm) and separation by one layer of lipids (2.25 nm). (B) Number of distinct clusters of CNTs (100 means each CNT is separate) according to the two different cutoff radii. (C) Number of CNTs in the biggest cluster *N*_max_ for a 2.25 nm cutoff (with fit to a power law) and average size N̅ of clusters. Note the double-logarithmic scale. (D) Cosine of the tilting angle averaged over all 100 CNTs.

Starting from a square grid of CNTs within a POPC lipid bilayer in a box of 70 nm width, the CNTs quickly arranged in clusters (Figures 1 and 2B). The size of the biggest cluster as a function of time follows a power law ∼*t*^0.65^ (Figure 2C). The clustered CNTs are more ordered in terms of their orientation, as can be seen from the decreasing tilting angle (Figure 2D). First, single CNTs met and formed small clusters that then fused. The process led to an increase in the number of next neighbors per CNT (Figure S7). We have shown previously^13^ that CNTs induce order in the lipids in their vicinity. By forming clusters, CNTs minimize the length of the interface between the highly ordered annular lipid shells around CNTs^13^ and bulk lipids. This interface is associated with a line tension and thus energetically costly.^45,46^ Due to depletion effects,^47^ clusters are also entropically favored. In contrast to classical Ostwald ripening,^48^ small clusters do not dissociate by releasing single CNTs on the time-scales of the simulations. Thus, fusion of small clusters, including single CNTs that have not encountered other CNTs yet, is the kinetically favored path to form large clusters.

In the clusters, CNTs remained separated by at least one lipid layer (a shared annular shell) and therefore are not in full contact (Figure 1 zoom-in and Figure 2A). Strong interactions between CNTs and lipid tails trap the lipids at the CNT surface. They lubricate the assembly process but also slow down the reorganization of existing clusters. The packing of CNTs separated by a single shared lipid shell gives rise to a pronounced peak in the radial distribution function at a CNT center-of-mass distance of about 2.1 nm (Figure 2A) and a strongly increased order parameter (see the Supporting Information). This shared lipid shell stabilizes the lipid-separated state.

### Cluster Diffusion

Cluster diffusion is a major determinant of CNT assembly, which relies on the collision and fusion of clusters. Large objects embedded in a membrane diffuse more slowly than small objects. The general dependence of the diffusion coefficient on the size of the diffusing object in a membrane can be calculated via the Saffman-Delbrück model^49,50^ for small inclusions. Diffusion for very large inclusions follows approximately a Stokes–Einstein relation.^51^ The transition between these behaviors in typical experiments occurs at a hydrodynamic radius of about 10 nm.^52,53^ A model that accurately covers the whole range was given by Hughes, Pailthorpe, and White,^54^ with a good approximation by Petrov and Schwille^55,56^ (HPW-PS). We calculated the diffusion coefficient for different effective radii of the clusters (details in the in the Supporting Information) and corrected the estimates for finite-size effects.^43,44^

**Figure 3:**
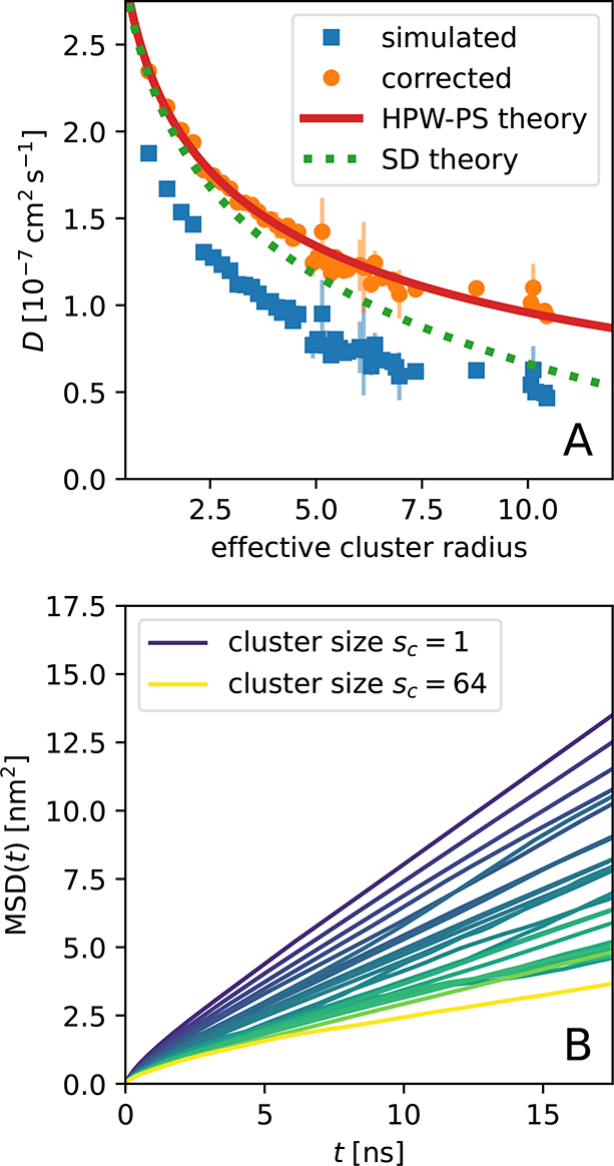
Dependence of diffusion coefficients of CNT clusters in the membrane on the effective cluster size. The size (*x*-axis) is given by the effective cluster radius  in units of nm, with *R*_CNT_ = 1.05 nm the CNT radius, and *s*_*c*_ the number of CNTs in a cluster. (A) Diffusion coefficients without (blue squares) and with correction for finite-size effects (orange circles). Curves show the Saffman-Delbrück model (SD theory; green dashes) and the Petrov-Schwille interpolation^55,56^ of the Hughes-Pailthorpe-White model (HPW-PS; solid red line). (B) Average mean squared displacements of the carbon nanotubes for different cluster sizes in one of the clustering simulations. Curves are colored from dark to light with increasing cluster size. We observed all cluster sizes from 1 to 20, all from 23 to 29 as well as 41 and 64. The window from 5 to 15 ns was used to extract cluster diffusion coefficients.

The finite-size-corrected diffusion coefficients from MD simulations quantitatively match the theoretical prediction of HPW-PS (Figure 3A). By contrast, the uncorrected values match the HPW-PS theory only poorly, giving a membrane viscosity of η_*m*_ = 7.2 × 10^–11^ Pa s m at a mean-squared error χ_ν_^2^ = 26.7 ± 0.2 (±1 would indicate a deviation of one standard error on average). From the fit after finite-size correction, we obtained an effective membrane viscosity of η_*m*_ = 4.5 × 10^–11^ Pa s m at χ_ν_^2^ = 1.1 ± 0.2. For comparison, we found η_*m*_ = 3.97 × 10^–11^ Pa s m for Martini simulations of pure POPC in earlier work.^44^ The slightly higher membrane viscosity here can be explained by the presence of the 100 CNTs that create hydrodynamic couplings and increase lipid order. We note that a shape-independent effective cluster radius (see in the Supporting Information) and a single constant value of the global membrane viscosity are sufficient to characterize the cluster-size dependence of the diffusion. Despite the drastic changes in membrane organization, the global viscosity does not change significantly during cluster formation. These methodological insights should help us to refine parametrization of mesoscale models^57^ for membrane inclusions (e.g., proteins) and the analysis of diffusion in crowded membranes.^58,59^

The match of our results with the theory for membrane inclusions shows that each CNT cluster behaves as one large object, even though the CNTs are not directly connected. The overall accordance of the corrected diffusion coefficients with the Hughes-Pailthorpe-White model confirms both the model itself and the finite-size correction procedure employed here, and it also allows us to extrapolate the diffusion behavior of CNT clusters or other membrane inclusions to larger scales.

### Conditions for Cluster Formation

#### CNT Properties

**Figure 4:**
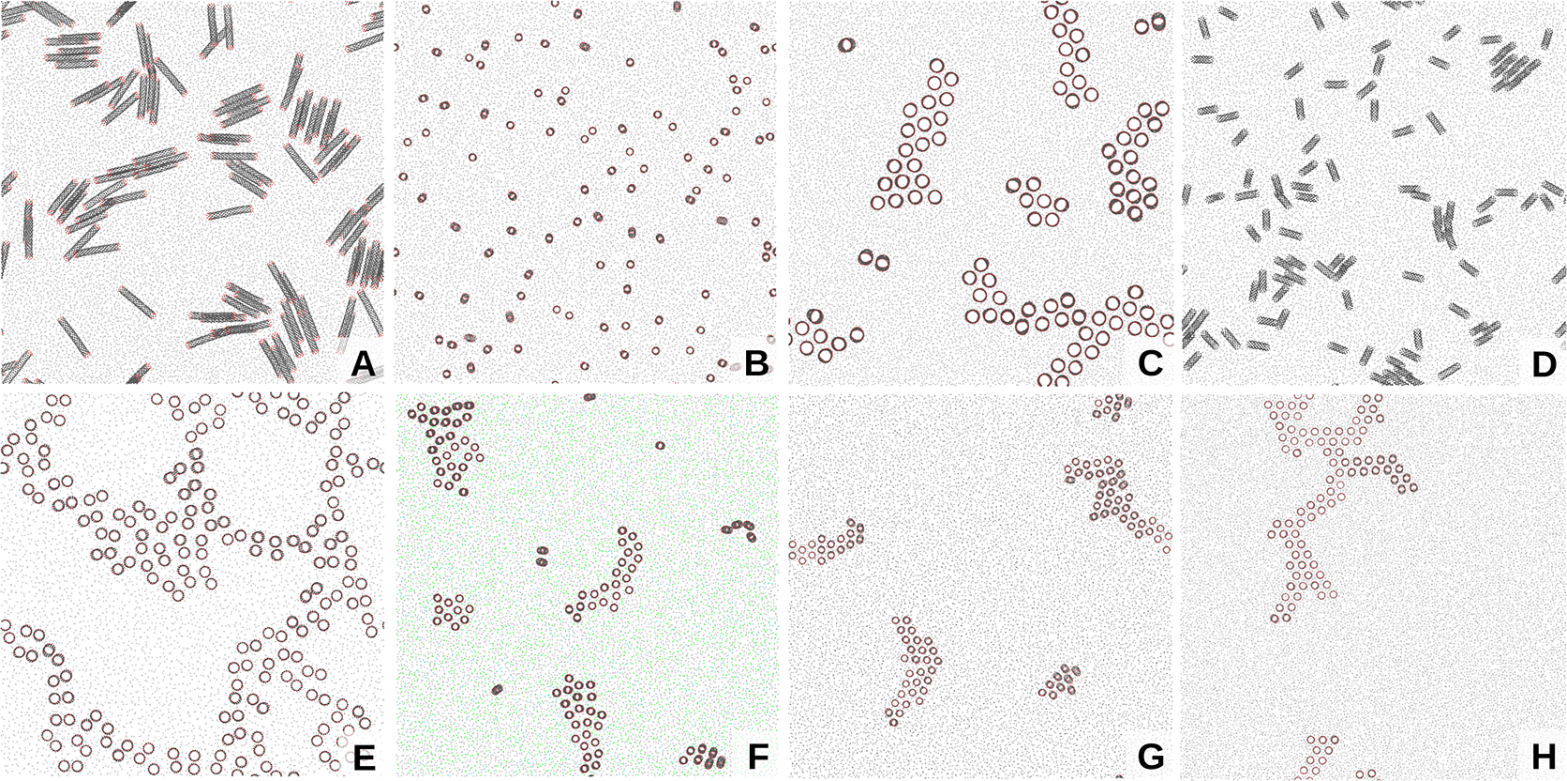
Snapshots from coarse-grained simulations of system setups with variations from the standard scheme (100 CNTs of 4.5 nm length with polar functionalization in POPC membranes). (A) CNTs longer than the thickness of the membrane. (B) CNTs shorter than the thickness of the membrane. (C) CNTs without polar end-groups. (D) CNTs with larger diameter. (E) 200 CNTs started as pairs. (F) Standard CNTs in POPG:POPE:cardiolipin mixture. (G) Standard CNTs at 350 K. (H) Standard CNTs in monoolein. For details of the simulation setups, see the Supporting Information, in particular Table S4.

The behavior of CNTs in the lipid membrane depends on their structural and chemical characteristics, as probed in exploratory simulations (see Tables S2–S7). CNTs that are significantly longer than the thickness of the membrane tilt to minimize the hydrophobic mismatch (Figures 4A and S4). This behavior is expected from previous simulations.^27,60^ In experiments, CNTs are usually coated by detergents or lipids. These coatings might prevent tilting and reduce the sensitivity of the assembly process to the length of the CNT.^26,61^ In the simulations, the shortest CNTs did not form stable clusters. Lipids bent over them and destroyed the order needed for controlled assembly (Figures 4B and S5). CNTs with larger diameter (Figure 4C) formed clusters with a more hexagonal structure. But in this case, there is a higher risk of lipids getting into the tube during preparation. Nonfunctionalized CNTs tilted to a horizontal position, often before they formed clusters (Figure 4D). These results show that the general behavior of the CNTs and therefore the process of cluster formation can be finely tuned.

In unbiased simulations, no spontaneous transition of two CNTs from a lipid-separated state to contact was observed. Therefore, we inserted pairs of CNTs that were already in full contact without being separated by lipids. These preassembled pairs stayed together over the whole simulation time (Figure 4E). This means that the contact state is at least metastable; but so is the lipid-separated state, as no additional direct contacts formed during the course of the simulation. In Markov chain Monte Carlo simulations of strongly hydrophobic transmembrane domains,^62−64^ the lipid-separated state was the minimum of the potential of mean force for strongly hydrophobic cylindrical inclusions. Dissipative-particle dynamics simulations of cylindrical membrane proteins,^60,65^ however, favored the contact state, also for end-functionalized carbon nanotubes in small lipid vesicles.^66,67^ These different results illustrate the subtle balance between direct and solvent-mediated interactions as well as entropic effects that govern the equilibrium and kinetics for transitions between lipid-separated states and contact states. These subtle interactions are challenging to capture in simulations and thus preclude firm predictions of the dominant state over very long time scales and in real systems.

Cluster formation depends on the hydrophobicity pattern of the CNT porin. We varied the size of the hydrophobic region of the carbon nanotubes by changing the number of rings of polar bead type at both ends of the CNT (Figure S1). We chose six configurations from completely hydrophobic (no polar rings at all; CNT functionalization type f0; see the Supporting Information) to five polar rings at each end (CNT type f5). The latter configuration has only two apolar rings in the middle of the tube, resulting in a strong negative hydrophobic mismatch. None of these CNTs in the different configurations made direct contacts with each other. Only for small hydrophobic regions at the end, full clustering was observed. The nonfunctionalized CNTs strongly tilted, which hampered further fusion. Cluster formation of CNTs with three or four polar rings soon saturated at about four to five CNTs per cluster. In this case, clusters rarely grew further and even disintegrated again. The strong order around the CNTs is thus necessary for the stability of large lipid-separated clusters.

#### Lipid Properties

As long as the length of the CNTs roughly matches the thickness of the membrane, the clustering behavior is only marginally affected by the specifics of diacylic lipids. Changing from pure POPC to a different lipid composition (POPE:POPG:Card. at a ratio 14:5:1, see Table S1 for lipid name abbreviations) did not change the behavior qualitatively (Figure 4F). By contrast, monoolein (MO) membranes sped up the clustering process by an order of magnitude and allowed for quicker reorganization to a hexagonal structure (Figures 4G and S7). This effect is similar to what is observed in simulations with POPC lipids at a higher temperature (350 K, Figure 4G).

We performed a systematic study for various different lipid types. For comparison with POPC lipid membranes, we considered lipids with short acyl chains (DLPC), lower saturation (DOPC), higher saturation (DPPC), a smaller headgroup (POPE), a net negative charge (POPG), and a single tail (MO). The cluster formation was similar for all lipids except for DLPC. The influence of the lipid headgroup is therefore negligible. The saturation of the tails also appears to play a comparably minor role, as the saturated palmytoyl tails in DPPC enhanced clustering only slightly compared to the monounsaturated oleoyl tails in DOPC. However, the length of the tails greatly influences clustering. The short tails of DLPC cause a hydrophobic mismatch that led to tilting of the CNTs as seen for overly long CNTs in POPC membranes. The consequence was a marked slowdown in the CNT assembly for DLPC lipids. The greatest speedup in assembly was observed for MO. It has only one acyl chain and therefore can rearrange more easily.

#### CNT–Lipid Interaction Strength

**Figure 5:**
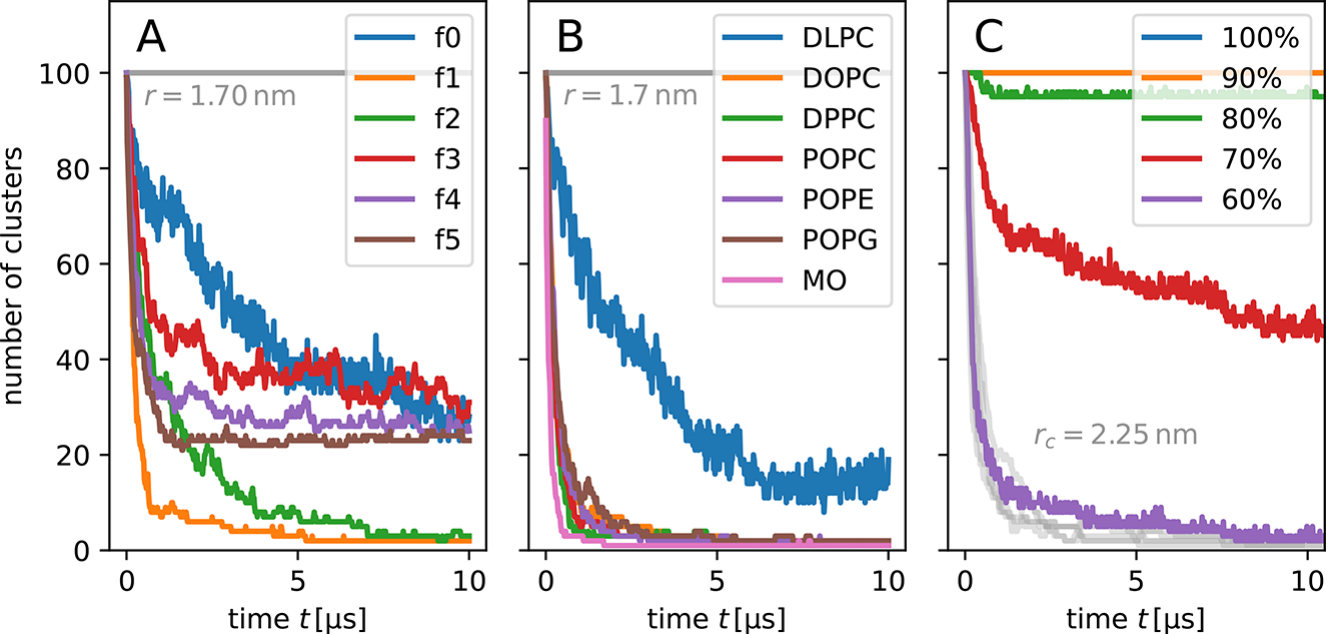
Dependence of CNT cluster formation on (A) hydrophobic mismatch for CNTs fX, with X the number of bead rings at each end that are polar, (B) lipid composition, and (C) CNT–lipid interaction strength. The number of clusters decreases as the CNTs merge into clusters of increasing size. See Figure S1 for details on the hydrophobicity patterns and Table S1 for the lipid name abbreviations.

Reducing CNT–lipid interactions facilitates the formation of direct contacts between CNTs (Figure 5C). We ran simulations in which we reduced the values of the Lennard-Jones energy parameters from 100% to 60% in steps of 10% for the CNT interactions with lipids and solvent, while keeping CNT–CNT interactions constant. Independent of the interaction strength, the clustering process (cutoff distance *r*_*c*_ = 2.25 nm between CNTs to identify clusters) was fast and saturated within the first few microseconds. However, the strength of the cross-interactions strongly influenced the formation of direct contacts (cutoff distance *r*_*c*_ = 1.7 nm between CNTs). For Lennard-Jones interactions reduced to 60% of the original values, almost all clusters were at close CNT–CNT contact; for 80% strength, almost no direct CNT–CNT contacts were observed, and none at all for 90% and 100%. A similar effect has been described for a simpler and more generic model of membrane inclusions.^62^ We conclude from these results that the strong cross-interactions between the CNTs and the lipid tails compared to the CNT–CNT interactions are the main cause for the lipid separation.

## Conclusions

Lipid-mediated self-assembly of CNTs promises to produce 2D hexagonal arrays with higher pore densities than in currently available fabrication techniques. Our simulations show that short, membrane-spanning CNTs have a strong tendency to aggregate into clusters in a lipid membrane. In our coarse-grained CNT model,^43^ the CNTs in these clusters retained an annular shell of lipids that prevented direct contact to the neighboring CNTs. Reasons are the comparably strong CNT–lipid interactions and the lipid order induced by the cylindrical geometry.^28,43^ Due to these strong interactions, the assembly of CNT clusters is dominated by the acyl chains whereas head groups have less effect. Especially monoolein led to a large speed-up compared to diacylic lipids. Cluster formation worked best with CNTs that carried polar end-functionalizations and whose length matched the thickness of the membrane. The process is sensitive to the tube length, whereas the diameter plays a minor role. For the assembly of longer tubes, the thicker membranes formed by diblock copolymers^68^ could be used instead of lipid membranes. In addition, solvent composition and conditions could be varied to manipulate the energetics and dynamics of the assembly process. We expect the most important solvent properties to be hydrophobicity and viscosity. An overly hydrophobic solvent could prevent the CNTs from inserting into the membrane while adding lipid coating or lipid-like detergents could facilitate the insertion process.^22,26^ According to the Saffman-Delbrück law, the viscosity of the fluid has an influence on the diffusivity of the membrane inclusions and thus influences cluster formation. A more viscous fluid slows down CNT diffusion but the dominant factor is the membrane viscosity, as we had shown in earlier work.^13^

Lipid-separated CNTs retained some degree of fluidity in nanoarrays. On the simulation time scale, contacts between the nanotubes did not form spontaneously for standard interactions.^27,34,43^ Such contacts only formed if CNT–lipid interactions were reduced. In standard simulations, already prepared contacts remained stable, which is indicative of a larger kinetic barrier between full-contact and lipid-separated states. It is not clear from simulations whether the lipid-separated clusters are stable or a metastable intermediate step toward crystallization. If the loose clusters were stable, it could lead to a new sort of material. The lipids would retain some fluidity and enhance the flexibility and biocompatibility, which are all desirable properties. If the lipid-separated state is only metastable, the delay in the transition to full CNT–CNT contact will be advantageous for assembly. A high concentration of CNTs could be reached before local nucleation cores can form that are mutually incompatible in their form or orientation. As a possible issue, areas of lipids can be encircled by a growing cluster and trapped inside. Such lipid islands could limit the maximum pore density. This might be overcome by chemically removing lipids from the membrane during cluster formation, e.g., by adding mild detergents.^69^

The diffusive dynamics of the clusters in the membrane is well-described by the Hughes-Pailthorpe-White extension by Petrov and Schwille^54,55^ of the Saffman-Delbrück model^49,50^ after accounting for finite-size effects in the MD simulations.^43,44,70^ The correct description of diffusive behavior will be important to estimate time scales for the assembly of large clusters, not only of CNTs but also of membrane proteins.^57^

In conclusion, our MD simulations establish the general feasibility of CNT–lipid-based nanomembranes with a high pore density. Due to the high flexibility concerning lipid properties, the possibilities to engineer suitable membranes for various applications are manifold.

## Supporting Information Available

Details on lipid names and simulation setup, parameters, and analysis; figures showing lipid order parameter, diffusion for different cluster sizes, simulation snapshots of differently sized CNTs, radial distribution of CNTs upon scaling of CNT–lipid interactions, and number of nearest neighbors in POPC and MO membranes plotted over simulation time (PDF)

## References

[ref1] Hummer G.; Rasaiah J. C.; Noworyta J. P. Water conduction through the hydrophobic channel of a carbon nanotube. Nature 2001, 414, 188–190. 10.1038/35102535. 11700553

[ref2] Majumder M.; Chopra N.; Andrews R.; Hinds B. J. Nanoscale hydrodynamics: Enhanced flow in carbon nanotubes (vol 438, pg 44, 2005). Nature 2005, 438, 930 10.1038/438930b. 16267546

[ref3] Holt J. K.; Park H. G.; Wang Y.; Stadermann M.; Artyukhin A. B.; Grigoropoulos C. P.; Noy A.; Bakajin O. Fast mass transport through sub-2-nanometer carbon nanotubes. Science 2006, 312, 1034–7. 10.1126/science.1126298. 16709781

[ref4] Rasaiah J. C.; Garde S.; Hummer G. Water in Nonpolar Confinement: From Nanotubes to Proteins and Beyond. Annu. Rev. Phys. Chem. 2008, 59, 713–740. 10.1146/annurev.physchem.59.032607.093815. 18092942

[ref5] Bocquet L.; Charlaix E. Nanofluidics, from bulk to interfaces. Chem. Soc. Rev. 2010, 39, 1073–1095. 10.1039/B909366B. 20179826

[ref6] Kalra A.; Garde S.; Hummer G. Osmotic water transport through carbon nanotube membranes. Proc. Natl. Acad. Sci. U. S. A. 2003, 100, 10175–80. 10.1073/pnas.1633354100. 12878724 PMC193535

[ref7] Menzl G.; Köfinger J.; Dellago C. Phase transition and interpore correlations of water in nanopore membranes. Phys. Rev. Lett. 2012, 109, 020602 10.1103/PhysRevLett.109.020602. 23030146

[ref8] Whitby M.; Quirke N. Fluid flow in carbon nanotubes and nanopipes. Nat. Nanotechnol. 2007, 2, 87–94. 10.1038/nnano.2006.175. 18654225

[ref9] Thomas M.; Corry B. A computational assessment of the permeability and salt rejection of carbon nanotube membranes and their application to water desalination. Philos. Trans. R. Soc. A 2015, 374, 1–20. 10.1098/rsta.2015.0020. PMC4696073 26712639

[ref10] Kocsis I.; Sun Z.; Legrand Y. M.; Barboiu M. Artificial water channels—deconvolution of natural Aquaporins through synthetic design. npj Clean Water 2018, 1, 13 10.1038/s41545-018-0013-y.

[ref11] Epsztein R.; DuChanois R. M.; Ritt C. L.; Noy A.; Elimelech M. Towards single-species selectivity of membranes with subnanometre pores. Nat. Nanotechnol. 2020, 15, 426 10.1038/s41565-020-0713-6. 32533116

[ref12] García-Fandiño R.; Sansom M. S. P. Designing biomimetic pores based on carbon nanotubes. Proc. Natl. Acad. Sci. U.S.A. 2012, 109, 6939–44. 10.1073/pnas.1119326109. 22509000 PMC3344987

[ref13] Vögele M.; Köfinger J.; Hummer G. Molecular dynamics simulations of carbon nanotube porins in lipid bilayers. Faraday Discuss. 2018, 209, 341 10.1039/C8FD00011E. 29974904

[ref14] Wu J.; Gerstandt K.; Zhang H.; Liu J.; Hinds B. J. Electrophoretically induced aqueous flow through single-walled carbon nanotube membranes. Nat. Nanotechnol. 2012, 7, 133–139. 10.1038/nnano.2011.240. 22245860 PMC4134328

[ref15] Li M.; Yang N.; Wood V.; Park H. G. Characterization of contact resistances in ceramic-coated vertically aligned carbon nanotube arrays. RSC Adv. 2019, 9, 7266–7275. 10.1039/C8RA10519G. 35548480 PMC9087477

[ref16] Liu L.; Yang C.; Zhao K.; Li J.; Wu H. C. Ultrashort single-walled carbon nanotubes in a lipid bilayer as a new nanopore sensor. Nat. Commun. 2013, 4, 1–8. 10.1038/ncomms3989. PMC3905707 24352224

[ref17] Kim K.; Geng J.; Tunuguntla R.; Comolli L. R.; Grigoropoulos C. P.; Ajo-Franklin C. M.; Noy A. Osmotically-driven transport in carbon nanotube porins. Nano Lett. 2014, 14, 7051–7056. 10.1021/nl5034446. 25372973

[ref18] Geng J.; Kim K.; Zhang J.; Escalada A.; Tunuguntla R.; Comolli L. R.; Allen F. I.; Shnyrova A. V.; Cho K. R.; Munoz D.; Wang Y. M.; Grigoropoulos C. P.; Ajo-Franklin C. M.; Frolov V. A.; Noy A. Stochastic transport through carbon nanotubes in lipid bilayers and live cell membranes. Nature 2014, 514, 612–615. 10.1038/nature13817. 25355362

[ref19] Ho N. T.; Siggel M.; Camacho K. V.; Bhaskara R. M.; Hicks J. M.; Yao Y.-C.; Zhang Y.; Köfinger J.; Hummer G.; Noy A. Membrane fusion and drug delivery with carbon nanotube porins. Proc. Natl. Acad. Sci. U.S.A. 2021, 118, e2016974118 10.1073/pnas.2016974118. 33941689 PMC8126853

[ref20] Raczyński P.; Górny K.; Pabiszczak M.; Gburski Z. Nanoindentation of biomembrane by carbon nanotubes - MD simulation. Comput. Mater. Sci. 2013, 70, 13–18. 10.1016/j.commatsci.2012.12.031.

[ref21] Wallace E. J.; Sansom M. S. Blocking of carbon nanotube based nanoinjectors by lipids: A simulation study. Nano Lett. 2008, 8, 2751–2756. 10.1021/nl801217f. 18665655

[ref22] Kraszewski S.; Picaud F.; Elhechmi I.; Gharbi T.; Ramseyer C. How long a functionalized carbon nanotube can passively penetrate a lipid membrane. Carbon 2012, 50, 5301–5308. 10.1016/j.carbon.2012.07.018.

[ref23] Kraszewski S.; Bianco A.; Tarek M.; Ramseyer C. Insertion of short amino-functionalized single-walled carbon nanotubes into phospholipid bilayer occurs by passive diffusion. PLoS One 2012, 7, 1–11. 10.1371/journal.pone.0040703. PMC3398044 22815794

[ref24] Lelimousin M.; Sansom M. S. P. Membrane perturbation by carbon nanotube insertion: Pathways to internalization. Small 2013, 9, 3639–3646. 10.1002/smll.201202640. 23418066

[ref25] Bhaskara R. M.; Linker S. M.; Vögele M.; Köfinger J.; Hummer G. Carbon Nanotubes Mediate Fusion of Lipid Vesicles. ACS Nano 2017, 11, 1273–1280. 10.1021/acsnano.6b05434. 28103440

[ref26] Choi M.-K.; Kim H.; Lee B.; Kim T.; Rho J.; Kim M.; Kim K. Understanding carbon nanotube channel formation in the lipid membrane. Nanotechnology 2018, 29, 115702 10.1088/1361-6528/aaa77b. 29332844

[ref27] Baoukina S.; Monticelli L.; Tieleman D. P. Interaction of Pristine and Functionalized Carbon Nanotubes with Lipid Membranes. J. Phys. Chem. B 2013, 117, 12113–12123. 10.1021/jp405732k. 24024494

[ref28] Garcia-Fandiño R.; Piñeiro Á.; Trick J. L.; Sansom M. S. P. Lipid Bilayer Membrane Perturbation by Embedded Nanopores: A Simulation Study. ACS Nano 2016, 10, 3693–3701. 10.1021/acsnano.6b00202. 26943498

[ref29] Kühlbrandt W. Two-dimensional crystallization of membrane proteins. Q. Rev. Biophys. 1992, 25, 1–49. 10.1017/S0033583500004716. 1589568

[ref30] Zhang Y.; Tunuguntla R. H.; Choi P.-o.; Noy A. Real-time dynamics of carbon nanotube porins in supported lipid membranes visualized by high-speed atomic force microscopy. Philos. Trans. R. Soc. B 2017, 372, 20160226 10.1098/rstb.2016.0226. PMC5483525 28630162

[ref31] Sullivan K.; Zhang Y.; Lopez J.; Lowe M.; Noy A. Carbon nanotube porin diffusion in mixed composition supported lipid bilayers. Sci. Rep. 2020, 10, 11908 10.1038/s41598-020-68059-2. 32681044 PMC7368039

[ref32] Marrink S. J.; Risselada H. J.; Yefimov S.; Tieleman D. P.; de Vries A. H. The MARTINI Force Field: Coarse Grained Model for Biomolecular Simulations. J. Phys. Chem. B 2007, 111, 7812–7824. 10.1021/jp071097f. 17569554

[ref33] Monticelli L.; Kandasamy S. K.; Periole X.; Larson R. G.; Tieleman D. P.; Marrink S. J. The MARTINI coarse-grained force field: Extension to proteins. J. Chem. Theory Comput. 2008, 4, 819–834. 10.1021/ct700324x. 26621095

[ref34] Monticelli L. On atomistic and coarse-grained models for C60 fullerene. J. Chem. Theory Comput. 2012, 8, 1370–1378. 10.1021/ct3000102. 26596752

[ref35] Bereau T.; Kremer K. Automated Parametrization of the Coarse-Grained Martini Force Field for Small Organic Molecules. J. Chem. Theory Comput. 2015, 11, 2783–2791. 10.1021/acs.jctc.5b00056. 26575571

[ref36] Periole X.; Huber T.; Marrink S.-j.; Sakmar T. P. G. Protein-Coupled Receptors Self-Assemble in Dynamics Simulations of Model Bilayers. J. Am. Chem. Soc. 2007, 129, 10126–10132. 10.1021/ja0706246. 17658882

[ref37] Koldsø H.; Shorthouse D.; Hélie J.; Sansom M. S. P. Lipid Clustering Correlates with Membrane Curvature as Revealed by Molecular Simulations of Complex Lipid Bilayers. PLoS Comput. Biol. 2014, 10, e1003911 10.1371/journal.pcbi.1003911. 25340788 PMC4207469

[ref38] Koldsø H.; Sansom M. S. P. Organization and Dynamics of Receptor Proteins in a Plasma Membrane. J. Am. Chem. Soc. 2015, 137, 14694–14704. 10.1021/jacs.5b08048. 26517394 PMC5591644

[ref39] Arnarez C.; Marrink S. J.; Periole X. Molecular mechanism of cardiolipin-mediated assembly of respiratory chain supercomplexes. Chem. Sci. 2016, 7, 4435 10.1039/C5SC04664E. 30155091 PMC6014297

[ref40] Fowler P. W.; Helie J.; Duncan A. L.; Chavent M.; Koldsø H.; Sansom M. S. P. Membrane stiffness is modified by integral membrane proteins. Soft Matter 2016, 12, 7792–7803. 10.1039/C6SM01186A. 27722554 PMC5314686

[ref41] Khelashvili G.; Albornoz P. B. C.; Johner N.; Mondal S.; Caffrey M.; Weinstein H. Why GPCRs behave differently in cubic and lamellar lipidic mesophases. J. Am. Chem. Soc. 2012, 134, 15858–15868. 10.1021/ja3056485. 22931253 PMC3469068

[ref42] Johner N.; Mondal S.; Morra G.; Caffrey M.; Weinstein H.; Khelashvili G. Protein and lipid interactions driving molecular mechanisms of in meso crystallization. J. Am. Chem. Soc. 2014, 136, 3271–3284. 10.1021/ja4129839. 24494670 PMC3985912

[ref43] Vögele M.; Hummer G. Divergent Diffusion Coefficients in Simulations of Fluids and Lipid Membranes. J. Phys. Chem. B 2016, 120, 8722–8732. 10.1021/acs.jpcb.6b05102. 27385207

[ref44] Vögele M.; Köfinger J.; Hummer G. Hydrodynamics of Diffusion in Lipid Membrane Simulations. Phys. Rev. Lett. 2018, 120, 268104 10.1103/PhysRevLett.120.268104. 30004782

[ref45] Akimov S. A.; Kuzmin P. I.; Zimmerberg J.; Cohen F. S.; Chizmadzhev Y. A. An elastic theory for line tension at a boundary separating two lipid monolayer regions of different thickness. J. Electroanalyt. Chem. 2004, 564, 13–18. 10.1016/j.jelechem.2003.10.030.

[ref46] Kuzmin P. I.; Akimov S. A.; Chizmadzhev Y. A.; Zimmerberg J.; Cohen F. S. Line tension and interaction energies of membrane rafts calculated from lipid splay and tilt. Biophys. J. 2005, 88, 1120–1133. 10.1529/biophysj.104.048223. 15542550 PMC1305117

[ref47] Asakura S.; Oosawa F. Interaction between particles suspended in solutions of macromolecules. J. Polym. Sci. 1958, 33, 183–192. 10.1002/pol.1958.1203312618.

[ref48] Ostwald W. Studien über die Bildung und Umwandlung fester Körper. Z. Phys. Chem. 1897, 22, 289–330. 10.1515/zpch-1897-2233.

[ref49] Saffman P. G.; Delbrück M. Brownian motion in biological membranes. Proc. Natl. Acad. Sci. U.S.A. 1975, 72, 3111–3113. 10.1073/pnas.72.8.3111. 1059096 PMC432930

[ref50] Saffman P. G. Brownian motion in thin sheets of viscous fluid. J. Fluid Mech. 1976, 73, 593 10.1017/S0022112076001511.

[ref51] Gambin Y.; Lopez-Esparza R.; Reffay M.; Sierecki E.; Gov N. S.; Genest M.; Hodges R. S.; Urbach W. Lateral mobility of proteins in liquid membranes revisited. Proc. Natl. Acad. Sci. U.S.A. 2006, 103, 2098–102. 10.1073/pnas.0511026103. 16461891 PMC1413751

[ref52] Guigas G.; Weiss M. Size-dependent diffusion of membrane inclusions. Biophys. J. 2006, 91, 2393–8. 10.1529/biophysj.106.087031. 16829562 PMC1562383

[ref53] Guigas G.; Weiss M. Influence of hydrophobic mismatching on membrane protein diffusion. Biophys. J. 2008, 95, L25–7. 10.1529/biophysj.108.136069. 18502792 PMC2479602

[ref54] Hughes B. D.; Pailthorpe B. A.; White L. R. The translational and rotational drag on a cylinder moving in a membrane. J. Fluid Mech. 1981, 110, 349 10.1017/S0022112081000785.

[ref55] Petrov E. P.; Schwille P. Translational diffusion in lipid membranes beyond the Saffman-Delbruck approximation. Biophys. J. 2008, 94, L41–L43. 10.1529/biophysj.107.126565. 18192354 PMC2242757

[ref56] Weiß K.; Neef A.; Van Q.; Kramer S.; Gregor I.; Enderlein J. Quantifying the diffusion of membrane proteins and peptides in black lipid membranes with 2-focus fluorescence correlation spectroscopy. Biophys. J. 2013, 105, 455–462. 10.1016/j.bpj.2013.06.004. 23870266 PMC3714877

[ref57] Chavent M.; Duncan A. L.; Rassam P.; Birkholz O.; Hélie J.; Reddy T.; Beliaev D.; Hambly B.; Piehler J.; Kleanthous C.; Sansom M. S. P. How nanoscale protein interactions determine the mesoscale dynamic organisation of bacterial outer membrane proteins. Nat. Commun. 2018, 9, 2846 10.1038/s41467-018-05255-9. 30030429 PMC6054660

[ref58] Guigas G.; Weiss M. Effects of protein crowding on membrane systems. Biochim. Biophys Acta Biomembranes 2016, 1858, 2441 10.1016/j.bbamem.2015.12.021. 26724385

[ref59] Javanainen M.; Martinez-Seara H.; Metzler R.; Vattulainen I. Diffusion of Integral Membrane Proteins in Protein-Rich Membranes. J. Phys. Chem. Lett. 2017, 8, 4308–4313. 10.1021/acs.jpclett.7b01758. 28823153

[ref60] Schmidt U.; Guigas G.; Weiss M. Cluster formation of transmembrane proteins due to hydrophobic mismatching. Phys. Rev. Lett. 2008, 101, 1–4. 10.1103/PhysRevLett.101.128104. 18851417

[ref61] Shen C.; Zou G.; Guo W.; Gao H. Lipid coating and end functionalization govern the formation and stability of transmembrane carbon nanotube porins. Carbon 2020, 164, 391–397. 10.1016/j.carbon.2020.04.011.

[ref62] West B.; Brown F. L. H.; Schmid F. Membrane-protein interactions in a generic coarse-grained model for lipid bilayers. Biophys. J. 2009, 96, 101–115. 10.1529/biophysj.108.138677. 18835907 PMC2710048

[ref63] Neder J.; West B.; Nielaba P.; Schmid F. Membrane-mediated Protein-protein Interaction: A Monte Carlo Study. Curr. Nanosci. 2011, 7, 656–666. 10.2174/157341311797483655.

[ref64] Neder J.; Nielaba P.; West B.; Schmid F. Interactions of membranes with coarse-grain proteins: A comparison. New J. Phys. 2012, 14, 1–24. 10.1088/1367-2630/14/12/125017.

[ref65] Morozova D.; Weiss M.; Guigas G. Shape as a determinant of membrane protein cluster formation. Soft Matter 2012, 8, 11905–11910. 10.1039/c2sm26720a.

[ref66] Dutt M.; Kuksenok O.; Nayhouse M. J.; Little S. R.; Balazs A. C. Modeling the self-assembly of lipids and nanotubes in solution: Forming vesicles and bicelles with transmembrane nanotube channels. ACS Nano 2011, 5, 4769–4782. 10.1021/nn201260r. 21604769

[ref67] Dutt M.; Nayhouse M. J.; Kuksenok O.; Little S. R.; Balazs A. C. Interactions of End-functionalized Nanotubes with Lipid Vesicles: Spontaneous Insertion and Nanotube Self-Organization. Curr. Nanosci. 2011, 7, 699–715. 10.2174/157341311797483772.

[ref68] Sanborn J. R.; Chen X.; Yao Y.; Hammons J. A.; Tunuguntla R. H.; Zhang Y.; Newcomb C. C.; Soltis J. A.; Yoreo J. J. D.; Buuren A. V.; Parikh A. N.; Noy A. Carbon Nanotube Porins in Amphiphilic Block Copolymers as Fully Synthetic Mimics of Biological Membranes. Adv. Mater. 2018, 30, e1803355 10.1002/adma.201803355. 30368926

[ref69] Lichtenberg D.; Ahyayauch H.; Alonso A.; Goñi F. M. Detergent solubilization of lipid bilayers: A balance of driving forces. Trends Biochem. Sci. 2013, 38, 85–93. 10.1016/j.tibs.2012.11.005. 23290685

[ref70] Vögele M.; Köfinger J.; Hummer G. Finite-Size-Corrected Rotational Diffusion Coefficients of Membrane Proteins and Carbon Nanotubes from Molecular Dynamics Simulations. J. Phys. Chem. B 2019, 123, 5099–5106. 10.1021/acs.jpcb.9b01656. 31132280 PMC6750896

