# Supplementary material for: Nanoporous Membranes of Densely Packed Carbon Nanotubes Formed by Lipid-Mediated Self-Assembly
Source: ACS Appl Bio Mater. 2022 Sep 7;7(2):528–34. doi: 10.1021/acsabm.2c00585 (PMC10880049; doi:10.1021/acsabm.2c00585)
Supplement: Supplementary file 1 — mt2c00585_si_001.pdf [file mt2c00585_si_001.pdf]

Supporting Information for: Nanoporous Membranes of Densely Packed Carbon Nanotubes Formed by Lipid-Mediated Self-Assembly

Martin Vögele,[†,‡] Jürgen Köfinger,[†] and Gerhard Hummer[*,†,¶]

†*Department of Theoretical Biophysics, Max Planck Institute of Biophysics, Max-von-Laue-Str. 3, 60438 Frankfurt am Main, Germany.*

‡*Current address: Schrödinger, Inc., 1540 Broadway, New York, NY 10036, USA*

¶*Institute for Biophysics, Goethe University Frankfurt, Max-von-Laue-Str. 1, 60438 Frankfurt am Main, Germany.*

E-mail: gerhard.hummer@biophys.mpg.de

Phone: +49 69 6303-2501

Lipid Names

We list the lipid types used in this work as well as their abbreviations in Table S1.

Table S1: Names and abbreviations of the lipid types used in this work.

abbreviation	full name
POPC	1-palmitoyl-2-oleoyl-sn-glycero-3-phosphocholine
DOPC	1,2-dioleoyl-sn-glycero-3-phosphocholine
DLPC	1,2-dilauroyl-sn-glycero-3-phosphocholine
DPPC	1,2-dipalmitoyl-sn-glycero-3-phosphocholine
POPE	1-palmitoyl-2-oleoyl-sn-glycero-3-phosphoethanolamine
POPG	1-palmitoyl-2-oleoyl-sn-glycero-3-phosphoglycerol
MO	2-monoolein
Card.	1,3-bis(sn-3'-phosphatidyl)-sn-glycerol

Simulation Setup

We simulated lipid bilayers containing upright carbon nanotubes under periodic boundary conditions in boxes with an edge length of 70 nm. Additional simulations were performed with smaller box sizes of about 50 nm to study certain parameters in more detail. Unless noted otherwise, the membranes consist of POPC lipids and contain 100 carbon nanotubes of 4.88 nm length (12 rings) with weakly polar end-groups. The shorter carbon nanotube models consist of only 8 rings of coarse-grained beads, the longer ones of 24 rings.

The hydrophobic thickness of CNTs of type fN with the default 12 rings was varied by changing N rings at either end from CNP to SNda bead type. The functionalization schemes in CNT variations f0 to f5 are shown in Figure S1.

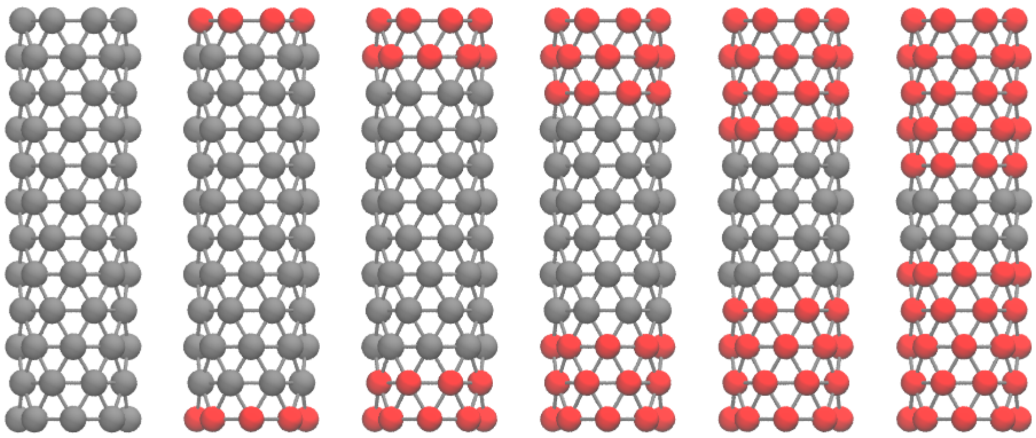

Figure S1: Variation of hydrophobic thickness by different numbers of polar rings. Beads of types CNP and SNda are shown in gray and red, respectively. Names of the configurations from left to right: f0, f1, f2, f3, f4, and f5.

Simulation Parameters

All simulations were run according to the same standard scheme unless noted otherwise. First, a steepest-descent energy minimization was performed for 15000 steps in order to remove steric clashes of the simulation beads. Then the system was equilibrated for 20 ns in an NPT ensemble with a timestep of 10 fs. The temperature was kept constant at 300 K by using a velocity rescaling thermostat[1] with a time constant of 1 ps. A pressure of 1 bar was established by semi-isotropic pressure coupling using a Berendsen barostat[2] with a time constant of 3 ps. In the data production runs, temperature coupling was achieved by the same thermostat as before and semi-isotropic pressure coupling (1 bar) by a Parrinello-Rahman barostat[3] with a time constant of $\tau_p = 12$ ps. The time step was set to 20 fs.

Table S2: Simulation parameters of CNT clustering simulations according to the standard scheme.

simulation	lipid type	num. of CNTs	funct. scheme	diameter [nm]	length [nm]	num. of lipids	solvent beads	box width [nm]	box height [nm]	duration [μs]
1	POPC	100	f1	1.20	4.88	14400	363000	68.75(7)	14.55(3)	14.40
2	POPC	100	f1	1.20	4.88	14400	363300	68.75(7)	14.56(3)	14.40
3	POPC	100	f1	1.20	4.88	14400	363200	68.76(7)	14.55(3)	14.40
4	POPC	100	f1	1.20	4.88	14400	363100	68.75(7)	14.55(3)	14.40
5	POPC	100	f1	1.20	4.88	14400	363900	68.76(7)	14.57(3)	14.40

Table S3: Simulation parameters of additional CNT clustering simulations. CNTs were started in only a part of the simulation box area to speed up CNT clustering. The first column provides the fraction of this area within the total area of the simulation box.

simulation	init. area	lipid type	num. of CNTs	funct. scheme	diameter [nm]	length [nm]	num. of lipids	solvent beads	box width [nm]	box height [nm]	duration [μs]
1	25.0%	POPC	100	f1	1.20	4.88	18200	408500	77.06(7)	13.47(2)	8.00
2	25.0%	POPC	100	f1	1.20	4.88	18200	408500	77.05(7)	13.47(2)	8.00
3	44.4%	POPC	100	f1	1.20	4.88	14400	336710	68.77(6)	13.81(3)	8.00
4	44.4%	POPC	100	f1	1.20	4.88	18200	408500	77.05(7)	13.47(2)	8.00
5	56.3%	POPC	100	f1	1.20	4.88	15406	338184	71.06(7)	13.23(2)	8.00
6	56.3%	POPC	100	f1	1.20	4.88	18200	408500	77.05(7)	13.47(2)	8.00

Table S4: Simulation parameters of individual exploratory CNT clustering simulations.

lipid type	num. of CNTs	funct. scheme	diameter [nm]	length [nm]	num. of lipids	solvent beads	box width [nm]	box height [nm]	duration [μs]
POPC	100	f1	1.20	9.77	14400	517700	70.23(14)	18.31(7)	9.60
POPC	100	f1	1.20	3.26	14400	372000	69.24(7)	14.52(3)	27.20
POPC	100	f1	2.39	4.88	14400	352300	71.29(11)	13.42(4)	8.00
POPC	100	f0	1.20	4.88	14400	363300	69.00(8)	14.46(3)	11.20
POPC	200	f1	1.20	4.88	14400	355200	69.90(7)	14.05(3)	24.00
PE/PG/Card.	100	f1	1.20	4.88	14200	363600	67.64(8)	15.06(4)	11.20
POPC (350 K)[a]	100	f1	1.20	4.88	14400	363000	72.01(10)	13.93(4)	11.20
MO	100	f1	1.20	4.88	22400	391600	66.78(11)	16.14(5)	10.00
MO	100	f1	1.20	4.88	22400	391600	66.77(11)	16.15(5)	10.00
MO	100	f1	1.20	4.88	22400	391200	66.76(12)	16.14(6)	10.00

[a] Temperature of 350 K.

Table S5: Simulation parameters of individual CNT clustering simulations with various lipid types.

lipid type	num. of CNTs	funct. scheme	diameter [nm]	length [nm]	num. of lipids	solvent beads	box width [nm]	box height [nm]	duration [μs]
POPC	100	f1	1.20	4.88	5400	154900	44.12(6)	15.17(5)	10.00
DOPC	100	f1	1.20	4.88	5400	155000	45.08(7)	14.82(5)	10.00
DLPC	100	f1	1.20	4.88	5400	164200	43.51(7)	15.34(5)	10.00
DPPC	100	f1	1.20	4.88	5400	159500	42.83(6)	16.11(4)	10.00
POPE	100	f1	1.20	4.88	5400	155100	43.13(6)	15.87(5)	10.00
POPG	100	f1	1.20	4.88	5400	150129	43.07(6)	15.69(5)	10.00
MO	100	f1	1.20	4.88	8000	164200	41.17(7)	17.49(6)	10.00

Table S6: Simulation parameters of CNT clustering simulations with CNTs of variant hydrophobic thickness.

lipid type	num. of CNTs	funct. scheme	diameter [nm]	length [nm]	num. of lipids	solvent beads	box width [nm]	box height [nm]	duration [μs]
POPC	100	f0	1.20	4.88	5400	155000	44.40(8)	14.98(5)	10.00
POPC	100	f1	1.20	4.88	5400	155000	44.12(6)	15.17(4)	10.00
POPC	100	f2	1.20	4.88	5400	154900	44.35(7)	15.00(5)	10.00
POPC	100	f3	1.20	4.88	5400	154900	44.72(6)	14.76(4)	10.00
POPC	100	f4	1.20	4.88	5400	155000	45.09(7)	14.52(5)	10.00
POPC	100	f5	1.20	4.88	5400	155100	45.27(8)	14.41(5)	10.00

Table S7: CNT clustering simulations with scaled CNT-lipid interactions.

interaction scaled to	lipid type	num. of CNTs	funct. scheme	diam. [nm]	length [nm]	num. of lipids	solvent beads	box width [nm]	box height [nm]	duration [μs]
100%	POPC	100	f1	1.20	4.88	5400	155100	43.23(5)	15.47(4)	40.00
90%	POPC	100	f1	1.20	4.88	5400	155100	43.35(6)	15.40(4)	42.60
80%	POPC	100	f1	1.20	4.88	5400	155100	43.42(5)	15.37(4)	38.00
70%	POPC	100	f1	1.20	4.88	5400	155100	43.48(6)	15.34(4)	48.00
60%	POPC	100	f1	1.20	4.88	5400	155100	43.60(6)	15.25(4)	38.00
50%	POPC	100	f1	1.20	4.88	5400	155100	43.66(6)	15.22(4)	39.67

Analysis

Tilting

Depending in particular on lipid type, CNT functionalization, and the resulting variations in the hydrophobic mismatch, we observed different tendencies for the CNTs to tilt with respect to the membrane normal. We quantified the tilting by calculating the distance vector between the centers of mass of the first and the last ring of coarse-grained beads of each carbon nanotube. The cosine of the tilting angle was determined as the ratio of its z component to its length. We averaged the cosine over all nanotubes as a measure of the tilting preference.

Radial Distribution of CNTs

In simulations in which the nanotubes did not tilt extensively, we treated them as points on a two-dimensional surface. For their centers of mass, we calculated an in-plane radial distribution function (RDF) that provides insight into how the CNTs arrange. To avoid artifacts from initial stages of cluster formation, we started RDF analysis after $8\,\mu$s of simulation time.

CNT Clustering

We used a connection-based clustering algorithm to group the CNTs according to their distance. From the results of the RDF, we deduced simple distance-based criteria for when to consider two tubes connected: one threshold distance for when they are in direct contact and another one for when they are separated by one lipid shell. We chose the separation between these two states to be between the first RDF maximum in simulations where direct contacts were observed, and the first one in simulations where no direct contacts were observed. As an example, we show the RDFs for the simulations with scaled CNT-CNT interactions in Figure S8.

Lipid Order Parameter

To quantify the order of the lipid tails, we used a coarse-grained order parameter, based on the angles α_j between each bond j connecting two Martini beads of one tail and the membrane normal. This order parameter

$$S^j_{\mathrm{Martini}} = \frac{1}{2}\left\langle 3\cos^2\alpha_j - 1\right\rangle \ . \tag{1}$$

indicates lipid order in a similar way as its atomistic counterpart but cannot be compared quantitatively. To obtain a single value per acyl chain, we averaged over all bonds in each chain. As in previous work,[4] we estimated the influence of the CNTs on the lipid ordering by assigning the order parameter of each lipid acyl chain (averaged over all atoms) to the annular lipid shell (defined by the minima of the RDF) to which the tail belongs. A separate category (S) was used for lipids that are shared between CNTs, i.e., in the first shell of two CNTs.

Cluster Diffusion

Diffusion Coefficient. We calculated the diffusion coefficient for different cluster sizes in the following way. We split the trajectories of the centers of mass of each CNT in blocks of 30 ns and calculated for each CNT in each block the in-plane mean squared displacement (MSD) with an efficient Fourier-based algorithm.[5] For each CNT and block, we also determined the average size of the cluster it belonged to as described above, and assigned the particular trajectory segment to that size. For each cluster size s_c, given by the number of CNTs a cluster contains, we then calculated the diffusion coefficient $\overline{D}_{s_c}$ by first averaging over the values of the individual MSDs assigned to it and then calculating one quarter of the slope of the averaged MSD with respect to time in the range from 5 ns to 15 ns.

In our error estimates, we accounted for the strong correlation in the translational diffusion of the CNTs within a cluster. We note that the relative motion of individual CNTs

within the cluster primarily affects the non-diffusive regime at the beginning of the MSD, where we do not fit. At longer times, the diffusion of the cluster as a whole dominates. To estimate $\overline{D}_{s_c}$ and its error, we calculated the values of the diffusion coefficient for each cluster size separately, i.e., the average will be the same as if we had calculated the average over the clusters first. However, we have to correct for the different number of independent values in the calculation of the standard error. Given estimates D_i^c ($i = 1, \ldots, n_c$) for the diffusion coefficients of the n_c clusters observed with exactly s_c CNTs, we define the corresponding standard error SEM_{s_c} via the unbiased sample variance σ^2

$$\mathrm{SEM}_{s_c}{}^2 = \frac{\sigma^2}{n_c} = \frac{1}{n_c(n_c-1)} \sum_{i=1}^{n_c} (D_i^c - \bar{D})^2 \tag{2}$$

with $\bar{D}$ the average. Due to the strong correlation, we now assume that for all CNTs α within a cluster i, the diffusion coefficients are identical to that of the cluster, $D_\alpha = D_i^c$. This allows us to rewrite the SEM as a sum over the $N = n_c s_c$ estimates D_α for CNTs in clusters of size s_c,

$$\mathrm{SEM}_{s_c}{}^2 = \frac{s_c^2}{N(N-s_c)} \sum_{\alpha=1}^{N} \frac{1}{s_c} (D_\alpha - \bar{D})^2 \tag{3}$$

The s_c from the sum cancels with the numerator of the prefactor and we arrive at the following expression for the standard error of the mean in terms of the diffusion coefficients D_α of individual CNTs belonging to clusters of size s_c:

$$\mathrm{SEM}_{s_c}{}^2 = \frac{s_c}{N(N-s_c)} \sum_{\alpha=1}^{N} (D_\alpha - \bar{D})^2 \,. \tag{4}$$

In our analysis, we only consider cluster sizes with more than one observation ($n_c > 1$).

Effective Cluster Radius. The area of one cluster is proportional to the number s_c of CNTs per cluster and thus its effective radius is proportional to $\sqrt{s_c}$. Approximating the

(highly non-circular) clusters as circular disks, we estimated an effective radius as

$$R_{\text{eff}} = R_{\text{CNT}}\sqrt{s_c} \tag{5}$$

with R_{CNT} being the effective radius of a single CNT. With lipids separating the CNTs in the clusters, we could use the geometric radius of a CNT. Instead, we used half the typical distance of two CNTs in the cluster, determined from the RDF as $R_{\text{CNT}} = \frac{1}{2}d_{\text{CNT}-\text{CNT}} = 1.05\,\text{nm}$.

Theoretical Models

We start by shortly presenting the different descriptions of membrane diffusion. The general dependence on the size of a diffusing object in a membrane is usually calculated via the Saffman-Delbrück (SD) model:[6,7]

$$D_{\text{SD}} = \frac{k_B T}{4\pi\mu_m h}\left(\ln\frac{1}{\varepsilon} - \gamma\right) \tag{6}$$

with k_B being the Boltzmann constant, T the temperature, and $\gamma \approx 0.5772$ Euler's constant. The variable ε denotes $R\mu_s/(h\mu_m)$, the hydrodynamic radius R scaled by the Saffman-Delbrück length. This has been contrasted by findings of a dependence that follows a Stokes-Einstein (SE) relation:[8]

$$D_{\text{SE}} = \frac{k_B T}{4\pi\mu_m h}\frac{\lambda}{R} \tag{7}$$

where λ was introduced as a characteristic length. Other data support the SD model.[9] Both models are limiting cases while the SD behavior occurs for small sizes and the SE behavior for large sizes.[10,11] A more accurate model over the whole range was given by Hughes, Pailthorpe and White[12] and a good approximation to it by Petrov and Schwille:[9,13]

$$D_{\text{HPW}} = \frac{k_B T}{4\pi\mu_m h}\left[(2\varepsilon - 1)\ln(\varepsilon) - \gamma + \frac{8\varepsilon}{\pi}\right]\left[1 + \frac{8\varepsilon^3}{\pi}\ln(\varepsilon) + \frac{c_1\varepsilon^{b_1}}{1 + c_2\varepsilon^{b_2}}\right]^{-1} \tag{8}$$

with the parameters $c_1 = 0.74819$, $b_1 = 2.74819$, $c_2 = 0.52119$, and $b_2 = 0.51465$. In the following, we investigate how accurately these models describe our data of carbon nanotubes during the cluster-formation process.

Model Comparison for Diffusion. We fitted the prediction by the Petrov-Schwille approximation of the Hughes-Pailthorpe-White model (HPW-PS theory) to the values obtained from the simulations. As we have seen, diffusion coefficients in membranes are subject to strong finite-size effects. We corrected for them by using an approximate correction formula.[14,15] In the fit, we fixed the viscosity $\eta_f = 10.2 \times 10^{-4}\,\mathrm{Pa\,s}$ of the Martini water at 300 K forming the solvent surrounding the membrane. However, the membrane is quite dense in CNTs. Therefore, we treated the membrane viscosity coefficient η_m as a free fit parameter. The fit was performed by numerically minimizing the reduced chi-squared error

$$\chi^2_\nu = \frac{1}{\nu} \sum_{s_c} \left(\frac{D_{0,s_c}(D_{\mathrm{PBC},s_c}, \eta_m) - D_{\mathrm{HPW},s_c}(R_{\mathrm{eff},s_c}, \eta_m)}{\mathrm{SEM}_{s_c}} \right)^2 \tag{9}$$

where the index s_c runs over all observed cluster sizes, D_{0,s_c} are the finite-size corrected diffusion coefficients calculated from the simulation values D_{PBC,s_c},[14,15] D_{HPW,s_c} are the theoretical predictions according to the HPW-PS model, SEM_{s_c} are standard errors, and ν are the number of degrees of freedom of the fit. A minimal value $\chi^2_\nu \gg 1$ indicates a deviation from the model not explained by the uncertainties while $\chi^2_\nu \ll 1$ indicates overfitting.

Detailed Results

Here we present additional figures to show detailed results of our simulations.

- Lipid order parameter: Figures S2 and S3
- Diffusion for different cluster sizes: Figures S4 and S5
- Differently sized CNTs: Figures S6 and S7
- Variation of CNT-CNT interactions: Figures S8
- Number of nearest neighbors: Figures S9

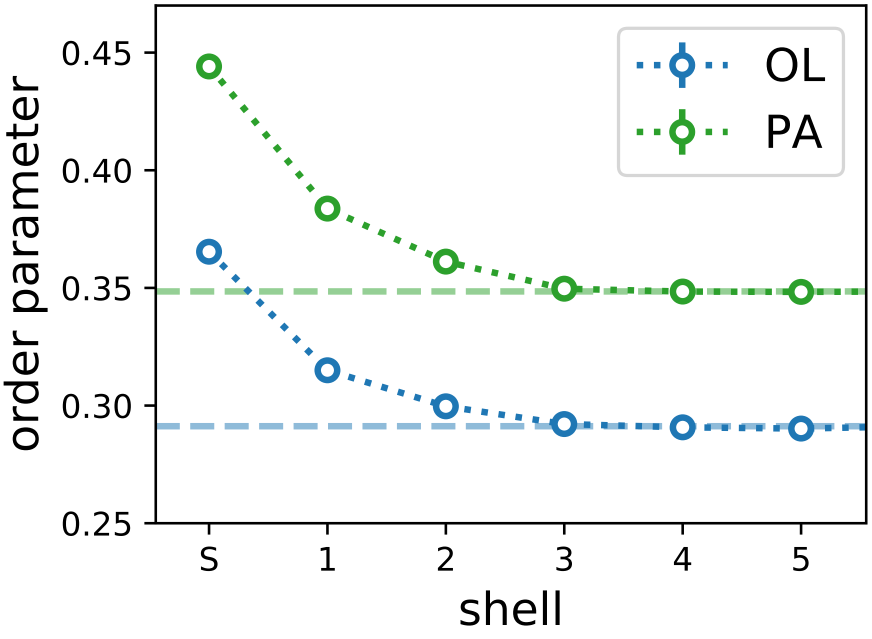

Figure S2: Order parameter of the POPC lipid chains (OL - oleyl, PA: palmitoyl) depending on the order of the shell around the nearest CNT. "S" indicates that a tail is in the first shell of two or more CNTs.

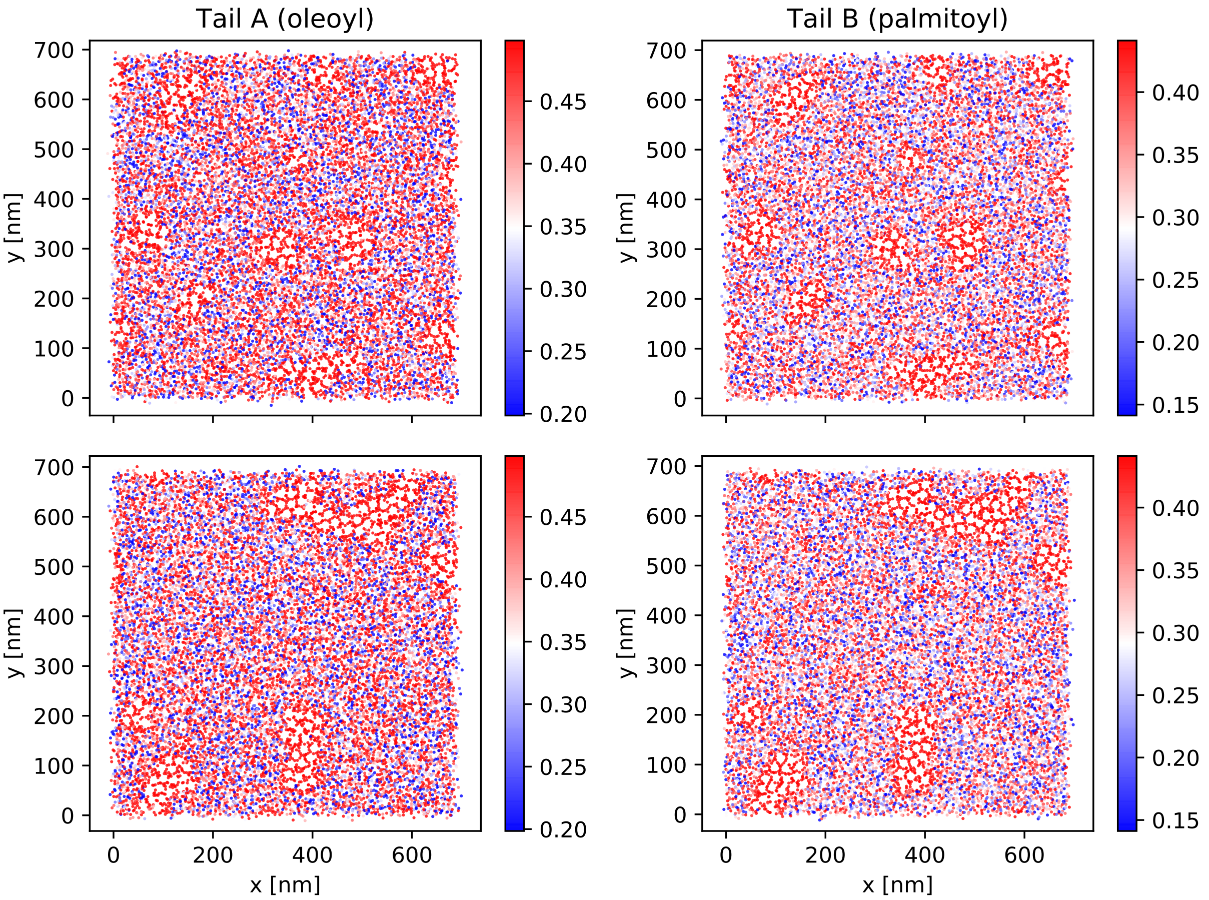

Figure S3: Example 2D snapshot of the order parameter of the POPC lipid chains after 4 μs of simulation (top) and after 12 μs of simulation (bottom). Color scales are centered around the respective average bulk value.

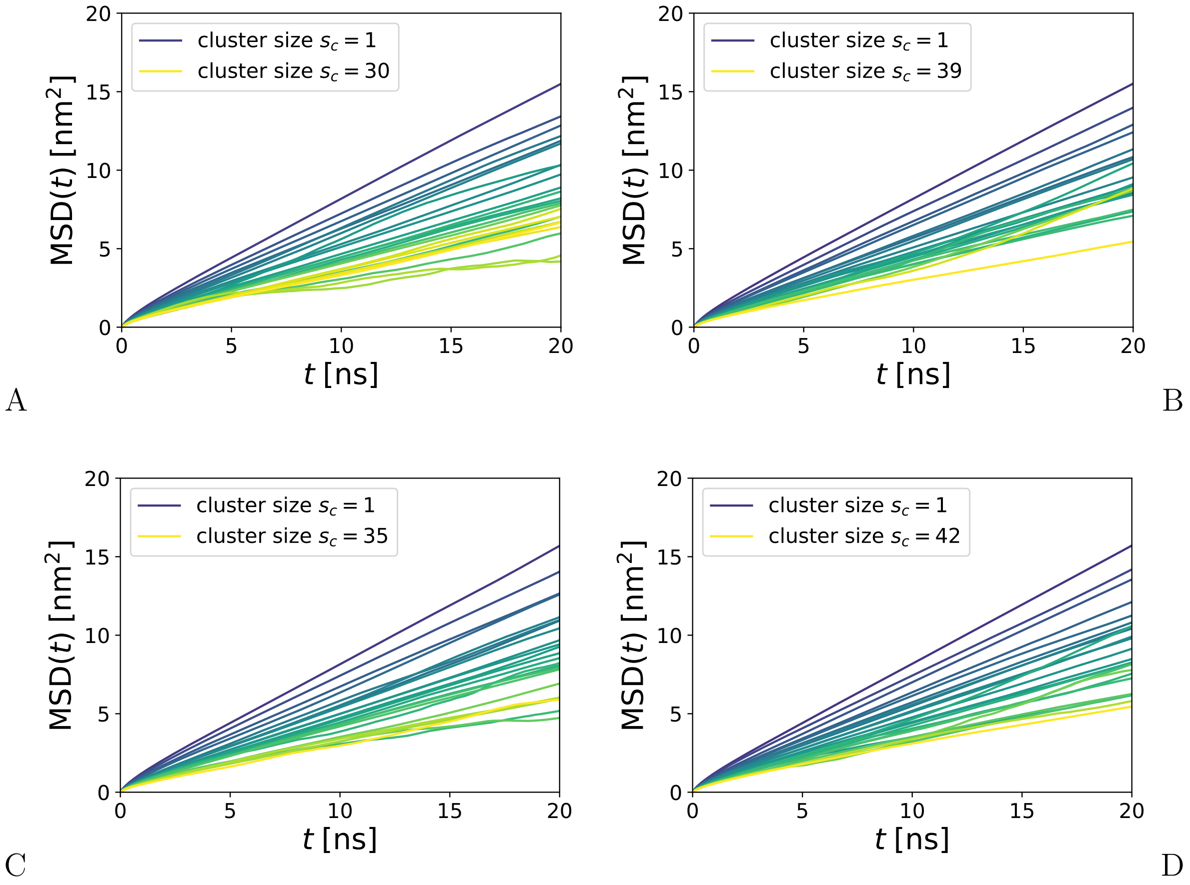

Figure S4: Average mean squared displacements of the carbon nanotubes for different cluster sizes for the simulations (A) 2, (B) 3, (C) 4, and (D) 5 from Table S2. Increasing cluster size is indicated by the purple-blue-green-yellow color gradient. Simulation 1 is shown in Fig. 3 in the main text.

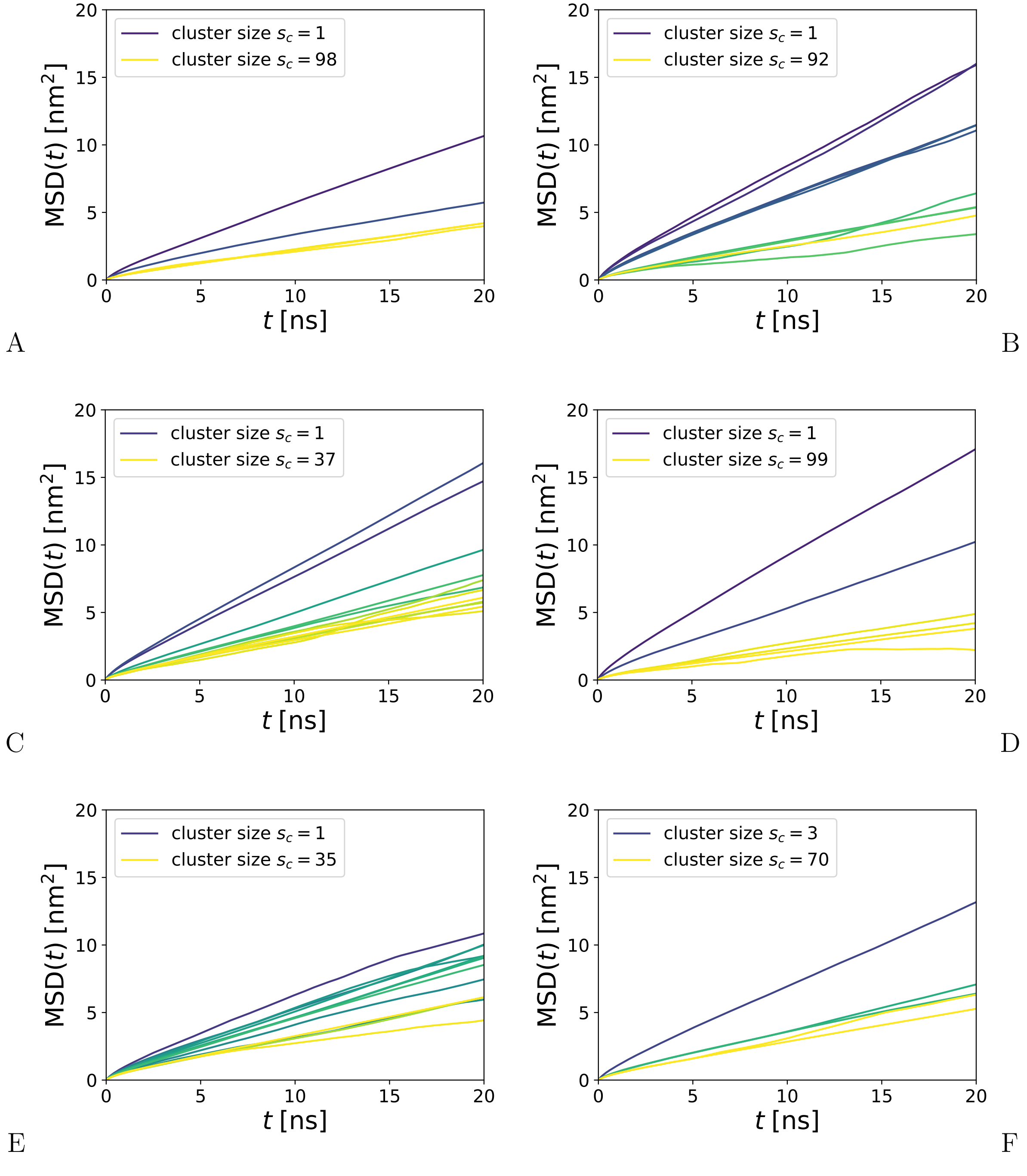

Figure S5: Average mean squared displacements of the carbon nanotubes for different cluster size for the simulations (A) 1, (B) 2, (C) 3, (D) 4, (E) 5, and (F) 6 from Table S3. Increasing cluster size is indicated by the purple-blue-green-yellow color gradient.

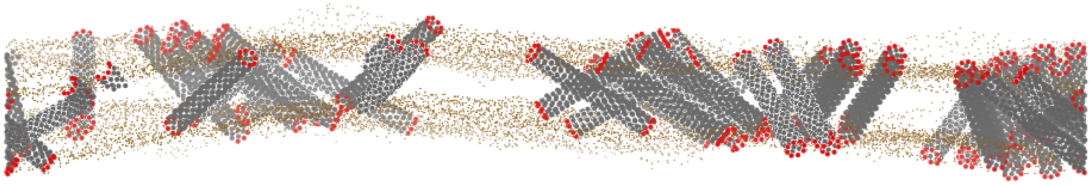

Figure S6: Long carbon nanotubes in a POPC membrane. Isolated and loosely interacting CNTs mostly tilt into the membrane. In larger, more organized clusters, the CNTs become more upright.

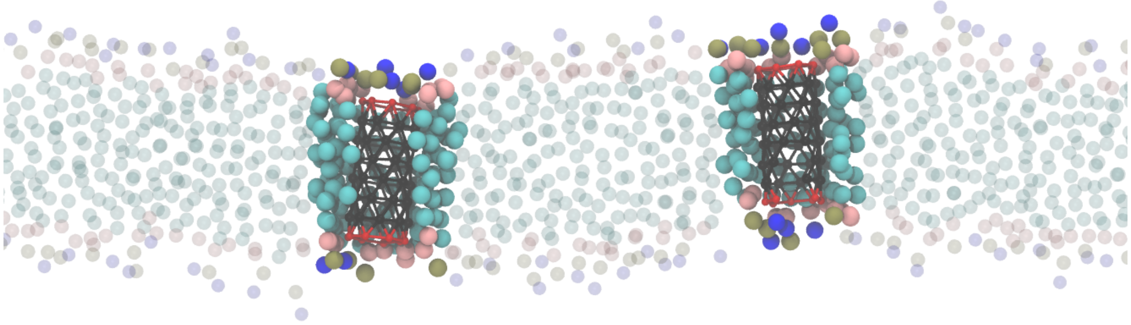

Figure S7: Short carbon nanotubes in a POPC membrane. Short CNTs do not form stable clusters. Driven by hydrophobic mismatch with respect to the membrane thickness, adhered lipids bend over the rim and apparently destroy the order needed for controlled assembly.

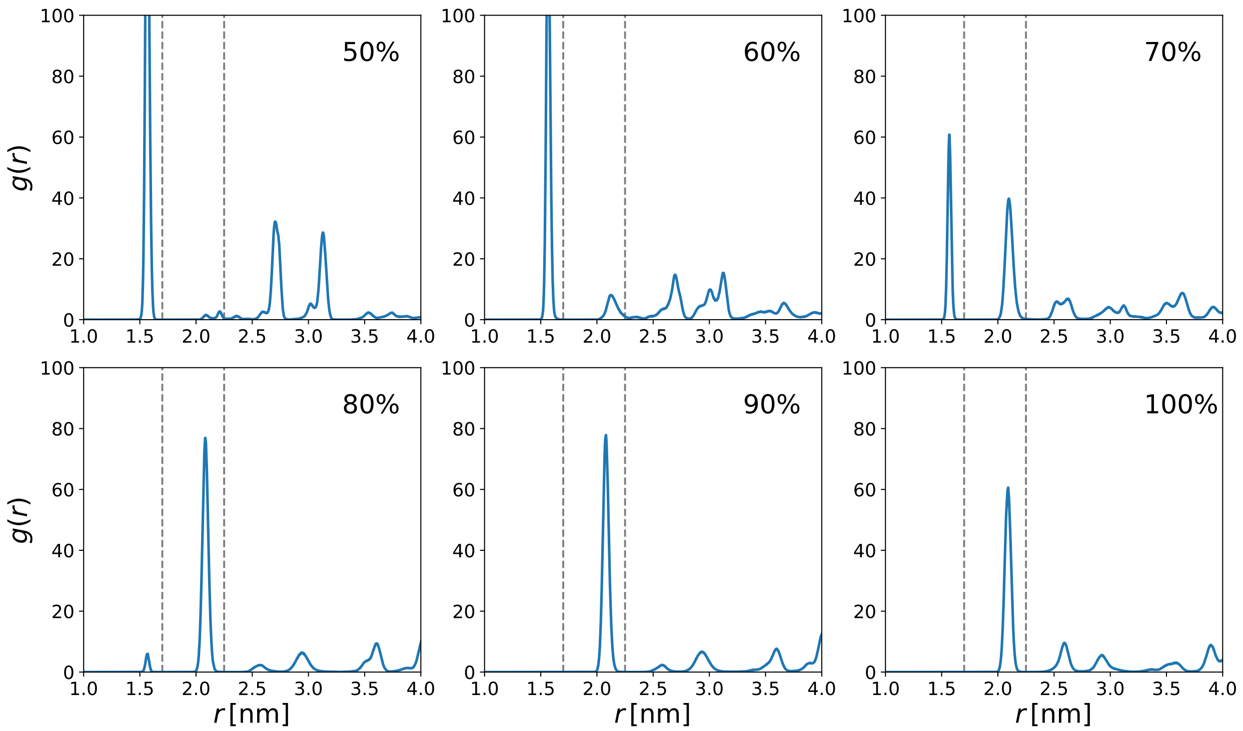

Figure S8: CNT-CNT radial distribution functions in simulations of different strengths of CNT-lipid interactions. Vertical dashed lines indicate the cut-off radii for close contacts (1.7 nm) and for lipid-separated contacts (2.25 nm).

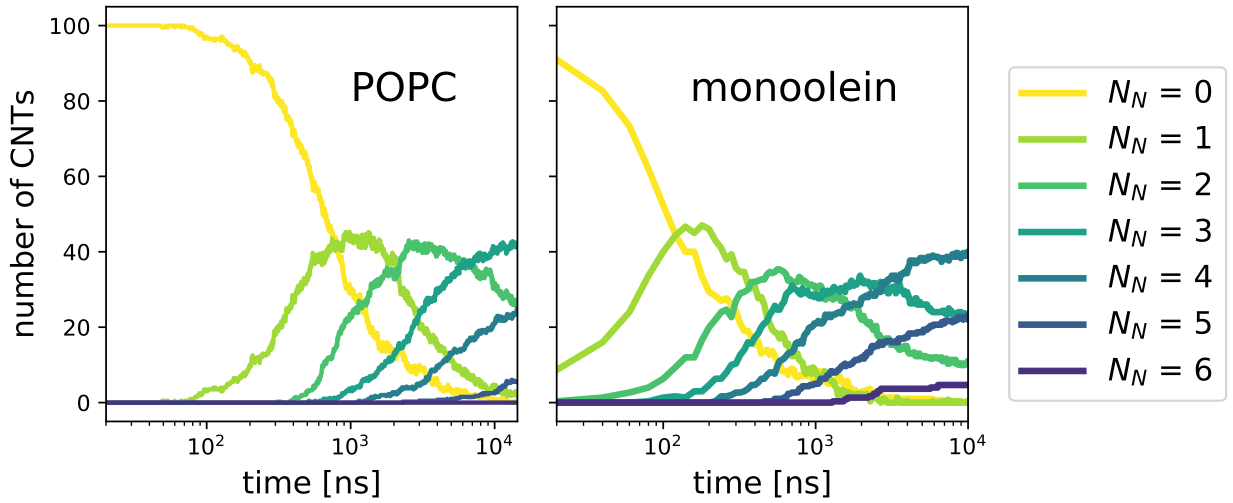

Figure S9: Number of nearest CNT neighbors in POPC and monoolein.

References

(1) Bussi, G.; Donadio, D.; Parrinello, M. Canonical sampling through velocity rescaling. *J. Chem. Phys.* **2007**, *126*, 014101.

(2) Berendsen, H. J. C.; Postma, J. P. M.; van Gunsteren, W. F.; DiNola, A.; Haak, J. R. Molecular dynamics with coupling to an external bath. *J Chem Phys* **1984**, *81*, 3684–3690.

(3) Parrinello, M.; Rahman, A. Polymorphic transitions in single crystals: A new molecular dynamics method. *J Appl Phys* **1981**, *52*, 7182–7190.

(4) Vögele, M.; Köfinger, J.; Hummer, G. Molecular dynamics simulations of carbon nanotube porins in lipid bilayers. *Faraday Discuss.* **2018**, *209*, 341.

(5) Calandrini, V.; Pellegrini, E.; Calligari, P.; Hinsen, K.; Kneller, G. R. nMoldyn-Interfacing spectroscopic experiments, molecular dynamics simulations and models for time correlation functions. *Collection SFN* **2011**, *12*, 201–232.

(6) Saffman, P. G.; Delbrück, M. Brownian motion in biological membranes. *Proc. Natl. Acad. Sci. U.S.A.* **1975**, *72*, 3111–3113.

(7) Saffman, P. G. Brownian motion in thin sheets of viscous fluid. *J. Fluid Mech.* **1976**, *73*, 593.

(8) Gambin, Y.; Lopez-Esparza, R.; Reffay, M.; Sierecki, E.; Gov, N. S.; Genest, M.; Hodges, R. S.; Urbach, W. Lateral mobility of proteins in liquid membranes revisited. *Proc. Natl. Acad. Sci. U.S.A.* **2006**, *103*, 2098–102.

(9) Weiß, K.; Neef, A.; Van, Q.; Kramer, S.; Gregor, I.; Enderlein, J. Quantifying the diffusion of membrane proteins and peptides in black lipid membranes with 2-focus fluorescence correlation spectroscopy. *Biophys. J.* **2013**, *105*, 455–462.

(10) Guigas, G.; Weiss, M. Size-dependent diffusion of membrane inclusions. *Biophys. J.* **2006**, *91*, 2393–8.

(11) Guigas, G.; Weiss, M. Influence of hydrophobic mismatching on membrane protein diffusion. *Biophys. J.* **2008**, *95*, L25–7.

(12) Hughes, B. D.; Pailthorpe, B. A.; White, L. R. The translational and rotational drag on a cylinder moving in a membrane. *J. Fluid Mech.* **1981**, *110*, 349.

(13) Petrov, E. P.; Schwille, P. Translational diffusion in lipid membranes beyond the Saffman-Delbruck approximation. *Biophys. J.* **2008**, *94*, L41–L43.

(14) Vögele, M.; Hummer, G. Divergent Diffusion Coefficients in Simulations of Fluids and Lipid Membranes. *J. Phys. Chem. B* **2016**, *120*, 8722–8732.

(15) Vögele, M.; Köfinger, J.; Hummer, G. Hydrodynamics of Diffusion in Lipid Membrane Simulations. *Phys. Rev. Lett.* **2018**, *120*, 268104.
